# Traits, strategies, and niches of liana species in a tropical seasonal rainforest

**DOI:** 10.1007/s00442-021-04937-4

**Published:** 2021-05-23

**Authors:** Qi Liu, Frank J. Sterck, Jiao-Lin Zhang, Arne Scheire, Evelien Konings, Min Cao, Li-Qing Sha, Lourens Poorter

**Affiliations:** 1 Forest Ecology and Forest Management Group, Wageningen University and Research, P.O. Box 47, 6700 AA Wageningen, The Netherlands; 2 CAS Key Laboratory of Tropical Forest Ecology, Xishuangbanna Tropical Botanical Garden, Chinese Academy of Sciences, Yunnan, 666303 China

**Keywords:** Environmental gradients, Lianas, Plant strategies, Plant traits, Tropical seasonal rainforest

## Abstract

Plant functional traits and strategies hold the promise to explain species distribution, but few studies have linked multiple traits to multiple niche dimensions (i.e., light, water, and nutrients). Here, we analyzed for 29 liana species in a Chinese tropical seasonal rainforest how: (1) trait associations and trade-offs lead to different plant strategies; and (2) how these traits shape species’ niche dimensions. Eighteen functional traits related to light, water, and nutrient use were measured and species niche dimensions were quantified using species distribution in a 20-ha plot combined with data on canopy gaps, topographic water availability, and soil nutrients. We found a tissue toughness spectrum ranging from soft to hard tissues along which species also varied from acquisitive to conservative water use, and a resource acquisition spectrum ranging from low to high light capture and nutrient use. Intriguingly, each spectrum partly reflected the conservative–acquisitive paradigm, but at the same time, the tissue toughness and the resource acquisition spectrum were uncoupled. Resource niche dimensions were better predicted by individual traits than by multivariate plant strategies. This suggests that trait components that underlie multivariate strategy axes, rather than the plant strategies themselves determine species distributions. Different traits were important for different niche dimensions. In conclusion, plant functional traits and strategies can indeed explain species distributions, but not in a simple and straight forward way. Although the identification of global plant strategies has significantly advanced the field, this research shows that global, multivariate generalizations are difficult to translate to local conditions, as different components of these strategies are important under different local conditions.

**Supplementary Information:**

The online version contains supplementary material available at 10.1007/s00442-021-04937-4.

## Introduction

Plant functional traits are morphological, physiological or phenological properties that affect plant growth, survival, and reproduction (Ackerly 2003), and hold the promise to explain plant species distribution patterns (McGill et al. 2006). Plant traits can be closely associated for biophysical reasons (e.g., larger leaves require more robust stems for mechanical support), because of allocation trade-offs (e.g., plants can invest limiting resources either in above or belowground organs), and/or because they reflect adaptations to similar environmental conditions. Trait associations, therefore, reflect ecological strategies of species to successfully complete their lifecycle in a specific environment (Grime 1974; Reich et al. 2003). Compared to the many studies that have evaluated trait spectra on broad spatial scales in the field (e.g., Wright et al. 2004) and within local communities (e.g., Fortunel et al. 2012), less studies have actually evaluated how traits affect plant performance in the field (but see, Poorter and Bongers 2006; Guimarães et al. 2018; Poorter et al. 2018), and even fewer studies have explicitly linked multiple traits to multiple dimensions of the plant niche (Sterck et al. 2011). Here, we evaluate for 29 liana species how leaf, stem, and root traits are associated, and how this shapes their light, water, and nutrient niche dimensions in a tropical seasonal rainforest.

Plant ecological strategies can explain the success of different species under different environmental conditions (Grime 2006; Westoby and Wright 2006). Since resource capture, use, and release are fundamental for plant functioning and performance, Díaz et al. (2004, 2016) proposed that, globally, plants show a trade-off between resource acquisition and conservation. For example, species with high specific leaf area and leaf nutrient concentrations can attain high photosynthetic rates and have the potential to be successful in high light environments, whereas species with higher tissue density and toughness can attain a longer life span, and therefore, persist in low light conditions (Wright and Westoby 2002; Poorter et al. 2006). These trait trade-offs are also known as the leaf economics spectrum (Wright et al. 2004). Similarly, species with cheap, soft stem wood and wide vessels can attain a higher hydraulic conductivity, gas exchange and growth, and can, therefore, achieve a competitive advantage in high resource environments (Santiago et al. 2004; van der Sande et al. 2019). By contrast, species with a high wood density are more resistant to drought-induced cavitation, mechanical damage, and pathogen attack, and can better survive under low resource conditions (Poorter et al. 2008; Cornwell et al. 2009).

Many studies have shown that for these reasons leaf, stem, and root traits are closely coupled (e.g., Reich et al. 2003; Freschet et al. 2010). However, leaf and hydraulic traits are also observed to be decoupled, with leaf economics spectrum corresponding to light capture and tissue longevity, and the hydraulics spectrum to water use and leaf temperature maintenance (Li et al. 2015). Similarly, root traits may be decoupled from leaf and stem traits, as roots have to deal with the acquisition of many different water and nutrient resources, and can obtain these resources in different ways, through mycorrhizae, nitrogen-fixing bacteria, or root exudates (Weemstra et al. 2016).

The species niche is defined as the n-dimensional hypervolume of environmental and biotic conditions under which a species can grow and reproduce (Hutchinson 1957), and therefore, reflects multiple niche dimensions. Henceforth, we often use the word “niche” to refer to one of the specific dimensions of the niche (i.e., nutrients, water, or light). Although spatial distribution patterns might also emerge from dispersal limitation (Hubbell 2001), niche theory predicts that species can differ in their distribution when they occupy places with distinct environmental conditions, are functionally different, and specialized for those conditions (Hutchinson 1957). Global plant strategies in resource capture and use (i.e., the acquisitive–conservative continuum, or the fast-slow continuum), are thought to explain species distribution and niches (Grime 1974; Díaz et al. 2004). Indeed, differential species distributions have been related to different species tolerances to shade (Sterck et al. 2006), drought (Engelbrecht et al. 2007; Condit et al. 2013), and nutrient stress (Baltzer and Thomas 2010). The idea is that the same conservative trait values allow plants to occupy low resource niches everywhere (Reich 2014), which has rarely been tested, because most studies have quantified only one component of the multidimensional resource niche (either water, light, or nutrients), but rarely the combination. Similarly, it is assumed that the whole trait package determines the niches, but it can be that different components of these global strategies are relevant for different dimensions of the resource niches. This study explores, therefore, the importance of traits and plant strategies for different dimensions (i.e., light, water, and nutrients) of the resource niche.

We focus on lianas (woody vines) as our study system. Lianas are an important component of tropical forest systems as they comprise up to 25% of the woody stems and 35% of the species, thus contributing substantially to forest structure and ecosystem functioning (Schnitzer 2015). Trait associations and trait-environment linkages may be different for lianas and trees. Since lianas are structural parasites, they may compete more efficiently for light (Estrada-Villegas and Schnitzer 2018), and may, therefore, show stronger trait associations with the light niche dimension than trees. Similarly, because lianas tend to have wide vessels, they are hydraulically more efficient, and stronger water spenders (van der Sande et al. 2019) and may, therefore, show stronger trait associations with the (topographic) water niche dimension than trees. By having acquisitive trait values, lianas would also have an advantage on fertile soils where they can attain high photosynthetic rates and rapid growth (Pasquini et al. 2015).

Here, we evaluated 18 leaf, stem, and root traits from 29 dominant liana species, and linked these to the light, water, and nutrient niche dimensions of the species in a permanent sample plot in a tropical seasonal rainforest of Xishuangbanna, China (Liu et al. 2021). We addressed the following questions and corresponding hypotheses:

First, how are liana traits associated? In line with the plant economics spectrum, we expect liana species to show a trait spectrum, ranging from trait values that increase resource acquisition and use to trait values that increase resource conservation (Reich 2014; Díaz et al. 2016). Since lianas are structural parasites, they allocate fewer resources towards structural support (Zhang et al. 2019) and this carbon can be partitioned to other functions. We expect liana stem traits to be weakly coordinated with leaf and root traits.

Second, how do functional traits determine the light, water, and nutrient niche dimensions of coexisting liana species? We predict that liana species with more acquisitive trait values generally occupy higher light, water, and nutrient resource niches. Hence, species with a high capacity for water and nutrient uptake and transport (i.e., high specific root length, wide stem vessel diameter and high leaf venation density), efficient leaf display (i.e., large leaf area and high specific leaf area), high leaf nutrient concentrations and fast gas exchange (i.e., high stomatal density, length, and pore index) will occupy high resource niches. In contrast, species with trait values that increase the persistence of roots, stems, and leaves (i.e., low specific root length, high wood density, high leaf thickness and dry matter content) will occupy low resource niches. We also expect that the light niche is best predicted by traits that reflect carbon construction costs and longevity (e.g., leaf and wood densities), that the water niche is best predicted by traits related to water transport (e.g., vessel diameter, leaf venation and stomata), and that the nutrient niche is best predicted by traits that reflect nutrient use (e.g., leaf nutrient concentrations and N:P ratio).

Third, which traits shape the abundance of liana species? We expect the most abundant species to be the one that is best adapted to the prevailing, most common environmental conditions in the plot. We expect the multivariate strategy to increase the performance under given environmental conditions, and hence, also under the prevailing environmental conditions. Since the tropical forest of Xishuangbanna is relatively humid and has a tall and closed canopy (Cao et al. 2006), we expect light to be the main limiting resource for the growth and survival of liana species, and that species with conservative traits related to carbon conservation and shade tolerance (i.e., high wood density and low specific leaf area) will be more abundant.

## Methods

### Study site

This study was conducted in the 20-ha Xishuangbanna Forest Dynamics Plot in Yunnan Province, Southwest China (21°37′ 08′′ N, 101°35′ 07′′ E). Mean annual rainfall is 1493 mm and mean annual temperature is 21.8 °C (Cao et al. 2006). The climate is shaped by warm-wet air masses from the Indian Ocean and continental air masses from the sub-tropical regions in summer and winter, which results in an alternation of dry and rainy seasons with a typical monsoon climate. The main soil type is laterite (Cao et al. 2006). The topography of the plot is heterogeneous with an elevational range from 709 to 869 m. The plot is trisected by three perennial streams that join in the Southwest of the plot (Lan et al. 2009). The vegetation in the area is a tropical seasonal rainforest with a canopy height of up to 60 m (Zhu 2006). The forest is dominated by *Parashorea chinensis* (Dipterocarpaceae), *Pittosporopsis kerrii* (Icacinaceae) and *Garcinia cowa* (Clusiaceae).

### Sample design

From 2013 to 2015, all rooted lianas with a diameter over 1 cm were tagged, mapped, and identified in the plot according to a standard method described by Gerwing et al. (2006) and Schnitzer et al. (2008). For species identification, plant samples were collected from October 2018 to December 2018. In total, the plot contains more than 136 liana species. For this study, we selected 29 of the most common liana species in the plot (with a density > 5 stems/ha) of which leaves and branches occurred below 8 m height so that they could be sampled. The 29 species comprised 71% of the total number of marked liana individuals. Lianas can be spatially aggregated due to their short-ranged dispersal (Clark et al. 2018). To avoid bias towards individuals thriving in high resource availability, we randomly selected 3–12 individuals throughout the plot (231 individual samples in total; Table S1). An approximately 40 cm long branch was sampled from the main stem between 3 and 8 m height, i.e., in the lower forest stratum. Only samples from healthy looking, sun-exposed and pest-free individuals with a diameter more than 1 cm were selected. In case the branch did not have enough leaves for all analyses, additional branches were collected from the same individual to obtain additional leaves. At the same time, the number of the 20 m × 20 m quadrats where the individual was rooted was recorded to link the individual in a later stage with light, soil nutrient, water, and topographic data.

### Functional trait measurements

For each individual, morphological and anatomical leaf, stem and root traits were measured (for full names of the traits, abbreviations, units, and major eco-physiological roles, see Table 1). These traits were selected because they represent the key traits of the leaf, stem, and root economic spectra (Wright et al. 2004; Chave et al. 2009; Díaz et al. 2016) and are potentially important for species’ growth rates and ecological strategies (Poorter et al. 2008; Wright et al. 2010).

**Table 1:** Overview of 18 functional traits studied: group of variable, name, abbreviation, unit, and major role in the plant

Trait name	Units	Abbreviation	Major role	Source
Leaf traits				
Leaf thickness	mm	LT	Increases physical leaf strength and path length for CO_2_ diffusion	Niinemets (1999)
Leaf area	mm^2^	LA	Increases light interception, carbon gain and water loss	Maharjan et al. (2011)
Specific leaf area	mm^2^ mg^−1^	SLA	Correlates positively with photosynthetic capacity and leaf turnover	Pérez-Harguindeguy et al. (2013)
Leaf dry matter content	mg g^−1^	LDMC	Correlates with leaf toughness	Pérez-Harguindeguy et al. (2013)
Leaf density	mg mm^−3^	LD	Dense leaves increase the resistance to CO_2_ diffusion and, hence, decrease photosynthetic carbon gain	Niinemets (1999)
Vein density	mm mm^−2^	VD	A structural determinant of hydraulic conductance and Photosynthetic rate	Pérez-Harguindeguy et al. (2013)
Stomatal density	no. mm^−2^	SD	Allows for a high supply of CO_2_ for assimilation, but increases transpiration	Tanaka and Shiraiwa (2009)
Stomatal length	μm	SL	Controls the exchange of gases—most importantly water vapor and CO_2_	Hetherington and Woodward (2003)
Stomatal pore index (SL^2^ × SD)	unitless	SPI	Increases leaf hydraulic conductance, photosynthesis, and transpiration	Sack et al. (2003)
Leaf nitrogen concentration	mg g^−1^	LNC	Increases the maximum photosynthetic rate, correlated with SLA	Pérez-Harguindeguy et al. (2013)
Leaf phosphorus concentration	mg g^−1^	LPC	Contributes to photosynthesis and other metabolic processes	Pérez-Harguindeguy et al. (2013)
Leaf potassium concentration	mg g^−1^	LKC	Contributes to stomatal coordination	Lines-Kelly (2000)
Leaf magnesium concentration	mg g^−1^	LMgC	Key component of chlorophyll and vital for photosynthesis	Lines-Kelly (2000)
Leaf zinc concentration	mg g^−1^	LZnC	Contributes to plant hormones responsible for stem and leaf expansion	Lines-Kelly (2000)
Leaf nitrogen to phosphorus ratio		N:P	Indicates whether N or P is limited to plant growth	Pérez-Harguindeguy et al. (2013)
Stem traits				
Wood density	g cm^−3^	WD	Positively correlates with strength (resistance to trunk breakage), mechanical safety, and cavitation resistances, and negatively correlates with growth rate	Van Gelder et al. (2006) Larjavaara and Muller-Landau (2010)
Vessel diameter	Μm	VesD	Increases water transport efficiency	Tyree et al. (1994)
Root traits				
Specific root length	m g^−1^	SRL	Increases potential nutrient and water uptake rates	Weemstra et al. (2016)

Trait measurements were made following protocols outlined by Pérez-Harguindeguy et al. (2013). For each individual, the branch and leaf samples were placed into a Ziplock bag with a moist paper towel to keep them hydrated. In the lab, leaves were separated from the branch, and three leaves were randomly selected. Leaf blade thickness (LT, mm) was measured at the central part of the leaf without major veins using a Syntek outside micrometer. Each leaf was scanned using a CanoScan 9000F Mark II scanner and then leaf area (LA, mm^2^) was calculated using ImageJ software (v.1.52a; Wayne Rasband, National Institutes of Health, USA; http://imagej.nih.gov/ij). Leaf water-saturated fresh mass was measured after leaves were immersed for 2 h in water. Leaves were subsequently oven-dried at 70 °C for leaf dry mass.

Stomatal density was measured using the impression method. We applied clear nail varnish to a 1 cm^2^ patch on the abaxial lamina immediately to the right of the mid vein (avoiding major veins). After 10 min, the nail polish was removed and mounted on a glass slide for making images under a microscope (Leica Microsystems Ltd., Leica DM2500, Germany). Four images were taken using 200–400× magnification, resulting in ca. 20–80 stomata per image, the scale was added at the same time. Stomatal density (SD, no. mm^−2^) of each leaf was measured by averaging the total number of stomata for each of the four images. Stomatal length (SL, mm) was measured for five randomly selected stomata on each image and was then averaged. The stomatal counts and measurements were conducted using the imaging software ImageJ (v.1.52a; Wayne Rasband, National Institutes of Health, USA; http://imagej.nih.gov/ij).

To measure the density of minor veins, for each species, several approximately 1 cm^2^ diamonds were excised from the central section of sample leaves and were kept in FAA solution [formalin:glacial acetic acid:ethanol (70%) = 5%:5%:90%] for storage. These leaf diamonds were immersed in a 5% NaOH solution and were heated in water bath at 65 °C. The solution was replaced once it turned dark until the veins were exposed. The samples were then washed with distilled water three times, and the diamonds that turned transparent were placed on glass slides and were stained with 1% safranin. For each leaf diamond, four images (with scale) were taken at 100× magnification using the microscope. The length of the minor veins within the view field was first traced and then measured using the ImageJ software (v.1.52a; Wayne Rasband, National Institutes of Health, USA; http://imagej.nih.gov/ij).

To measure leaf nutrient concentrations, fresh leaf samples were cleaned with a moist tissue and then oven-dried at 70 °C for at least 48 h, ground to a fine powder with a crusher, and then passed through a 60-mesh sieve. The powders were placed in plastic bags and sent to the Public Technology Service Center, Xishuangbanna Tropical Botanical Garden, Chinese Academy of Sciences. In the laboratory, leaf nitrogen concentration (LNC, mg g^−1^) was analyzed using an Elemental Analyzer (Vario MAX CN, Elementar Analysensysteme GmbH, Germany). An inductively coupled plasma atomic-emission spectrometer (iCAP7400, Thermo Fisher Scientific, Bremen, Germany) was used to measure leaf phosphorus concentration (LPC, mg g^−1^), leaf potassium concentration (LKC mg g^−1^), leaf magnesium concentration (LMgC, mg g^−1^) and leaf zinc concentration (LZnC, mg g^−1^).

Stem traits were determined from a 5-cm-long branch segment at the base of each collected branch. The bark and pith were excluded and then the fresh wood volume (cm^3^) was determined using the water displacement method. This branch segment was then dried in an oven at 70 °C for 72 h and then weighted for its dry weight (g).

An additional ca. 3 cm branch piece was sampled from the branch base for anatomical measurements and embedded in FAA. For each branch sample, four cross-section images were taken at 100–500× magnification using a microscope (Zeiss Smartzoom 5 Digital Microscope, Germany). For each original image, to avoid the threshold difference in ImageJ analysis, we first erased the vessel area with the eraser tool in Adobe Photoshop CS6, then imported to the ImageJ Software where measurements were finished automatically without color. To estimate the vessel diameter (VesD, μm), the ten widest vessel diameters from each of the four images were measured and averaged. We focused on the largest vessels because they are expected to contribute the strongest to hydraulic conductance (Tyree et al. 1994).

To sample liana roots, the roots were dug up from 0 to 20 cm soil depth. Most of the soil was removed by slightly shaking the roots. The root sample was then placed into a marked Ziplock bag with moist paper towels to keep the sample hydrated. The roots were then carefully cleaned and placed in a shallow tray of water and fully spread out using scissors and scotch tape. Since plant roots are often divided by root (ramification) order, the third branch order of fine roots (starting at the root tip) was cut for measurements, as this part of the roots are responsible for nutrient uptake (Fitter 1982). The root length was measured using a scanner (CanoScan 9000F Mark II) and was further analyzed with the WinRHIZO (Regent Instruments Inc., Quebec, Canada) root analysis program. The roots were oven-dried at 70 °C for 72 h and weighed for their dry mass.

### Functional trait calculations

Specific leaf area (SLA, mm^2^ mg^−1^) was calculated as the leaf area per unit leaf dry mass. Petioles were not included in the SLA calculation as they can be very large for rainforest species, and because they are more related to leaf positioning than biomass efficiency for leaf display. For compound leaves, SLA was based on all available leaflets, but the rachis was not included in the SLA calculation because rachis was much heavier than the sum of the leaflets. Leaf dry matter content (LDMC, mg g^−1^) was calculated as the leaf dry mass divided by the leaf water-saturated fresh mass. Leaf density (LD, g cm^−3^) is the leaf dry mass per unit leaf volume, and it was calculated as 1/(SLA × LT). The minor vein density (VD, mm mm^−2^) was calculated as the total length of minor veins per unit area. Stomatal pore index (SPI, unitless) was calculated as SD × SL2. Wood density (WD, g cm^−3^) was calculated as branch wood dry mass over branch fresh wood volume without bark. Specific root length (SRL, m g^−1^) was calculated as the root length per unit root dry mass.

### Soil nutrient niches

To quantify soil nutrient availability across the plot, soils were sampled in a systematic way to create soil nutrient maps. The soil was sampled in 2011 using a regular 30 m × 30 m grid throughout the 20-ha plot. Each of the 252 nodes in this grid was used as a “base point”. Together with each base point, two additional sampling points were added, located at random distances of 2 and 5 m, 2 and 15 m or 5 and 15 m along a random compass bearing away from the correlated base point. Hence, in total, 756 soil samples were taken. At each sample point, 500 g of topsoil at 0–10 cm depth was collected, as the topsoil layer is most nutrient rich, and plants obtain most soil nutrients from the topsoil. Fresh soil samples were placed in plastic bags, shipped to the Public Technology Service Center, Xishuangbanna Tropical Botanical Garden, Chinese Academy of Sciences for nutrient analysis (nitrogen, phosphorus, and potassium). Using this original soil data, an ordinary kriging was performed to generate a sub-quadrat grid map of 10 m × 10 m for each soil variable (Cressie 1992). For each 20 m × 20 m quadrat, soil nutrient concentrations were then calculated as the mean of the values at each of the nine nodes of the 10 m × 10 m sub-quadrats within that quadrat using the geoR package in the R (see Hu et al. 2012). For each species, the soil nutrient niche dimensions of soil N, P, and K were quantified as the average value of the soil nutrients of the quadrats where rooted individuals of the species were found.

### Water availability

Spatial topographic information was used to quantify the water availability. To describe the topography, the plot was subdivided into 500 20 m × 20 m quadrats. For each quadrat, the elevation and slope were measured following Harms et al. (2001): the elevation was calculated as the mean of the elevation at the four quadrat corners and the slope was based on the mean angular deviation from the horizontal of each of the four triangular planes formed by connecting three corners. We used the topographic wetness index (TWI) to describe the water niche of the species. For each species, the water niche was quantified as the average value of the TWI of quadrats where rooted individuals of the species were found. This index is defined as TWI = ln(α/tan β), where α is the local upslope area draining through a certain point per unit contour length and tan β is the local slope in radians (Beven and Kirkby 1979). The specific catchment area is a parameter describing the tendency of the site to receive water from upslope area and local slope is a parameter describing the tendency to evacuate water (Gruber and Peckham 2009). This index is, therefore, a relative measure of the long-term soil moisture availability of a given site in the landscape. High TWI indicates a strong water accumulation. In ArcGis Desktop 10.6.1, we used the Flow Accumulation tool to calculate α and the slope tool to calculate β. It should be said that TWI is a topographic measure of potential water availability. It would have been better to really measure soil water potential across the plot as it is also determined by soil depth and texture, but this is logistically challenging.

### Light niche

To quantify the light niche for all liana species, we inferred the light conditions for each quadrat from the forest structure. The quadrat (20 m × 20 m) was defined as a “gap” when its vertically projected open area was larger than 50% (i.e., > 200 m^2^) of the quadrat in 2014 (Nicholas 1982; Liu et al. 2014). In total, there were 31 gaps in the 20-ha plot. For each species, the percentage of individuals in the 20-ha plot that occurred in gaps was quantified as the species light niche. The binary measure of gaps (present in gaps or not) becomes, therefore, a continuous estimator of the light niche dimension, varying from 0% when no individuals are found in gaps, to 100% when all individuals are situated in gaps.

### Statistical analyses

The variation in environmental factors encountered in the studied forest is visualized by relative frequency diagrams showing our observations for all quadrats (Fig. S1). For the analysis, we compiled a dataset with species mean values for all measured plant traits (Table 1). All statistical tests were conducted using RStudio R 3.6.2 (R Core Team 2019). Eight traits (LA, SLA, SD, SL, LKC, LMgC, LZnC, and VesD) were ln-transformed to achieve normality. Since SRL data were missing for 4 species, we used the package “missMDA” to impute missing values, thus yielding a dataset of 18 functional traits for 29 liana species.

To evaluate how traits were associated, we used pairwise Pearson’s correlation and a principal component analysis (PCA) using species mean values as data points. Since SLA plays a pivotal role in the leaf economics spectrum, an additional multiple regression was used to evaluate how SLA depends on its underlying components, LT and LD.

To evaluate which traits shape the species niche, we fitted all possible linear regression models that included combinations of 18 species’ mean traits using the dredge function of the MuMIn package (Barton 2012), with the 18 species’ mean traits as explanatory variables and the species niche as the dependent variable. To avoid multicollinearity, six variables (LA, LD, LDMC, SL, N_P, and WD) with a Variance Inflation Factor > 5 were excluded before model fitting (Zuur et al. 2010). We then selected the best-fitting models with delta Akaike information criterion (ΔAICc) < 2, and averaged the coefficients of the best-fitting models to obtain the most robust and conservative model coefficients (compared to using only the best-fitting model). We did so use the model.avg function of the MuMIn package in R (Sterck et al. 2014).

To evaluate whether multivariate trait strategies can provide a better prediction of the species niche, we quantified the species strategy as the species regression scores on the first and second axis of the trait PCA, and did a series of multiple regressions of the five species niches on the two principal components. We then compared their *R*^2^ with best models. The same analysis was conducted between liana relative abundance in the plot and functional traits. Relative abundance was calculated as the total individuals of each species divided by the total individuals of all liana species across the plot.

## Results

### Trait correlations

**Fig. 1:**
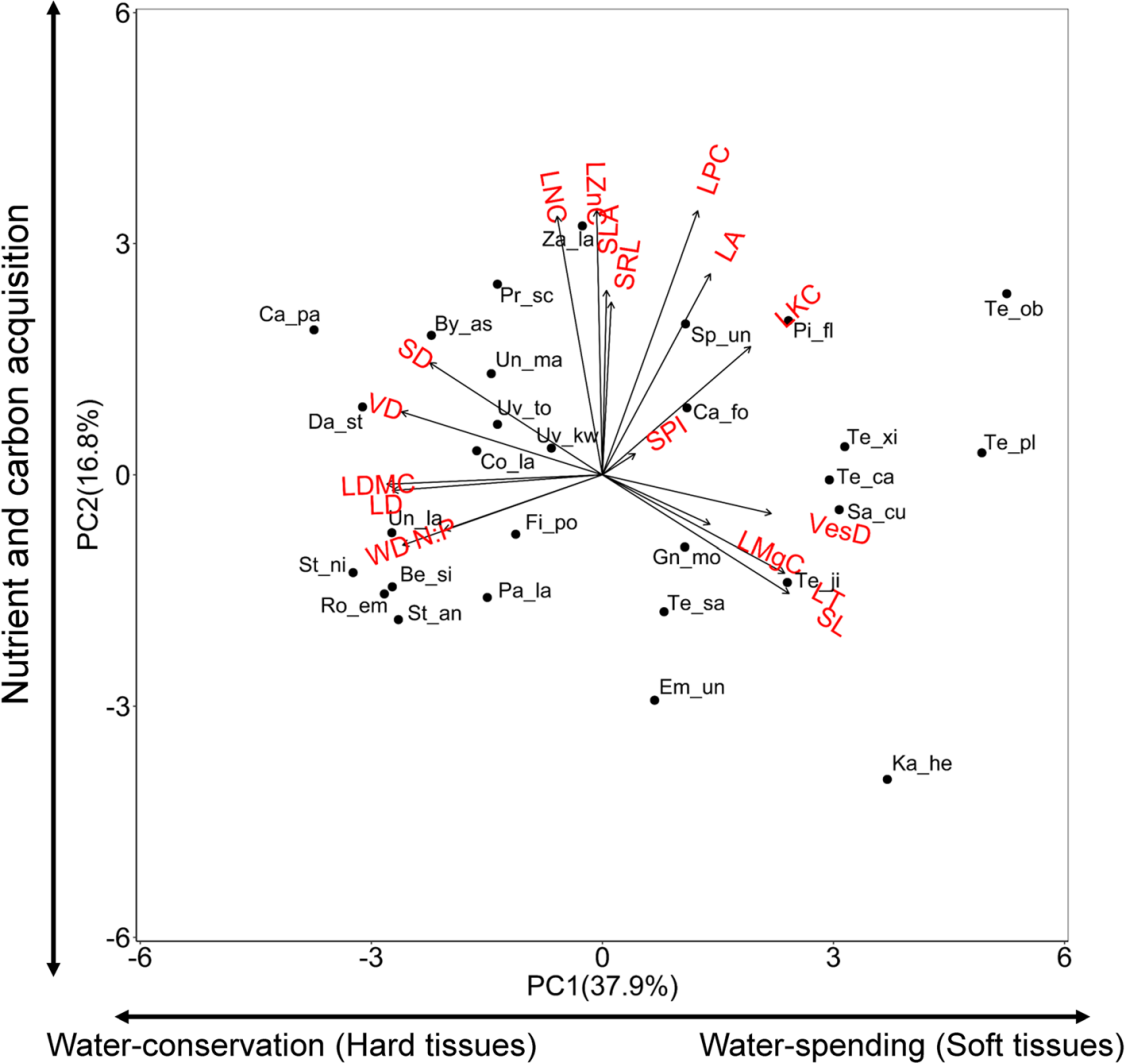
Principal component analysis (PCA) of multivariate trait associations of 18 traits for 29 liana species in a tropical seasonal rainforest in Xishuangbanna, China. Average species trait data were used as data points. The first two PCA axes and the loadings (indicated by arrows) of 18 traits were shown. The arrows at next to the *y*-axis and *x*-axis indicate the three spectra (tissue toughness spectrum, water use spectrum, and nutrient and carbon acquisition spectrum). Each point represents one species. For trait abbreviations, see Table 1. For loading scores and species code, see Table S1 and Table S2. Traits were normalized prior to analysis (see “Methods” section)

The first two PCA axes explained 55% of the variation and showed two spectra of trait variation. The first PCA axis ranged from liana species with water-conserving traits and tough tissues to the left (i.e., high leaf density, leaf dry matter content, wood density), to liana species with a more water-spending strategy and soft tissues (high vessel diameter and stomatal length) to the right (Fig. 1). Unexpectedly, leaf nutrient traits were not significantly associated with this axis. The second PCA axis showed changes across liana species in leaf nutrient concentration (Fig. 1), SRL and SLA. The second PCA axis reflected photosynthetic carbon gain and was mostly determined by SLA and LNC, and by LZnC and LKC, which are related to metabolism (Zn) and potential gas exchange (K).

**Fig. 2:**
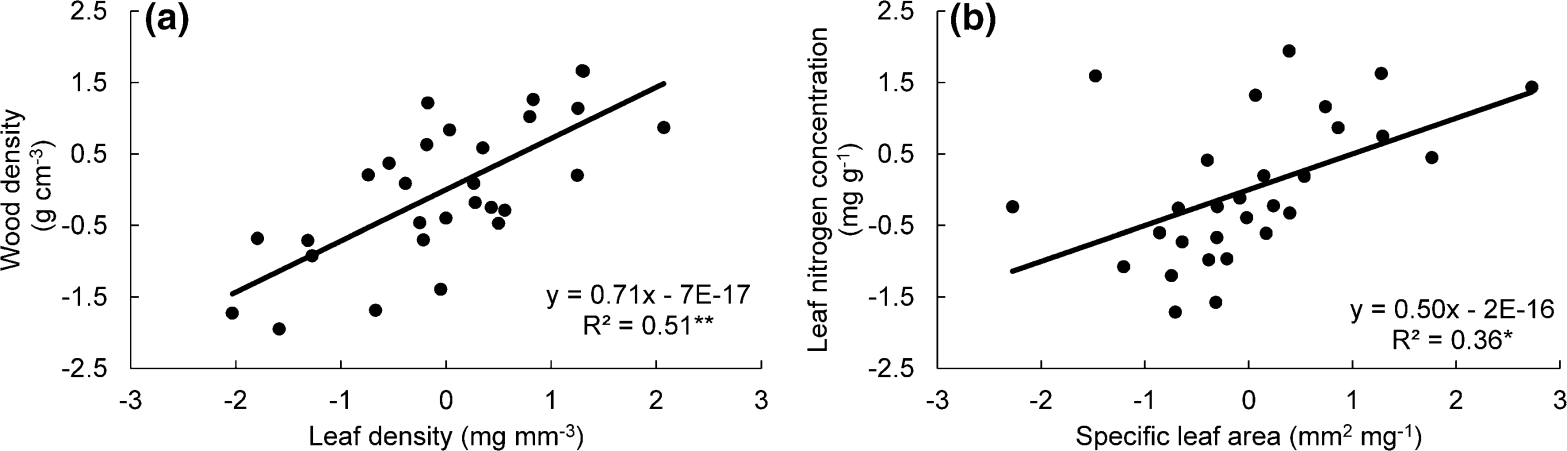
Relationships between **a** wood density and leaf density and **b** leaf nitrogen concentration and specific leaf area across 29 liana species in Xishuangbanna tropical seasonal rainforest. Regression lines, regression equations, R^2^ and significance level (*P < 0.05; **P < 0.01) are shown. Each dot indicates a species. Traits were normalized as described in the “Methods” section

Relationships between functional traits were analyzed among the 29 liana species (Table 2). Traits of the water-conservation spectrum (LDMC, LD, VD, and SD) were significantly positively correlated with each other and with stem trait wood density (WD) (Table 2 and Fig. 2a). The same trend was found for the water–acquisitive spectrum, with lianas possessing increased vessel diameter (VesD) having larger stomata (high SL). In this resource capture spectrum, SLA was positively associated with LNC (Fig. 2b). Surprisingly, specific root length (SRL) did not show any significant relationships with leaf and stem traits (Table 2). A multiple regression of ln(SLA) on ln(leaf thickness) and ln(leaf density) showed that SLA was equally determined by leaf thickness (standardized regression coefficient = − 1.07, *P* < 0.001) and leaf density (standardized regression coefficient = − 1.06, *P* < 0.001).

**Table 2:** Pearson’s correlation among 18 leaf, stem, and root traits (see Table 1 for trait abbreviations) of 29 liana species in the tropical seasonal rainforest of Xishuangbanna, China

	LT	LA	LD	LDMC	SLA	VD	SD	SL	SPI	LNC	LPC	LKC	LMgC	LZnC	N:P	WD	VesD
LA	0.26																
LD	− 0.71**	− 0.32															
LDMC	− 0.67**	− 0.3	0.91**														
SLA	− 0.45*	0.09	− 0.25	− 0.2													
VD	− 0.72**	− 0.14	0.76**	0.84**	0.08												
SD	− 0.50**	− 0.01	0.67**	0.67**	− 0.16	0.67**											
SL	0.58**	0.14	− 0.70**	− 0.66**	0.02	− 0.68**	− 0.87**										
SPI	0.10	0.31	− 0.09	− 0.03	− 0.07	0.01	0.21	0.27									
LNC	− 0.31	0.23	0.08	0.17	0.50**	0.16	0.19	− 0.26	− 0.02								
LPC	0.22	0.69**	− 0.31	− 0.26	0.19	− 0.24	− 0.05	0.09	0.12	0.57**							
LKC	0.44*	0.39*	− 0.549**	− 0.60**	0.04	− 0.54**	− 0.33	0.29	− 0.13	0.07	0.50**						
LMgC	0.32	0.12	− 0.29	− 0.50**	− 0.12	− 0.46*	− 0.24	0.35	0.18	− 0.33	− 0.09	0.52**					
LZnC	− 0.28	0.15	0	− 0.06	0.40*	0.09	0.22	− 0.32	− 0.17	0.41*	0.43*	0.35	0.13				
N:P	− 0.54**	− 0.57**	0.47**	0.49**	0.22	0.44*	0.29	− 0.37*	− 0.11	0.31	− 0.57**	− 0.46*	− 0.11	− 0.09			
WD	− 0.48**	− 0.58**	0.72**	0.75**	− 0.19	0.58**	0.57**	− 0.63**	− 0.19	0.13	− 0.40*	− 0.42*	− 0.27	− 0.14	0.66**		
VesD	0.51**	0.31	− 0.57**	− 0.61**	0	− 0.59**	− 0.55**	0.60**	0.17	− 0.12	0.21	0.16	0.16	− 0.15	− 0.41*	− 0.73**	
SRL	− 0.03	0.24	− 0.08	− 0.17	0.13	0.11	0.27	− 0.18	0.24	0.17	0.11	0.25	0.03	0.33	− 0.01	− 0.18	− 0.05

Significance levels (*P < 0.05; **P < 0.01) were shown

### Functional traits as predictors of resource niches

**Fig. 3:**
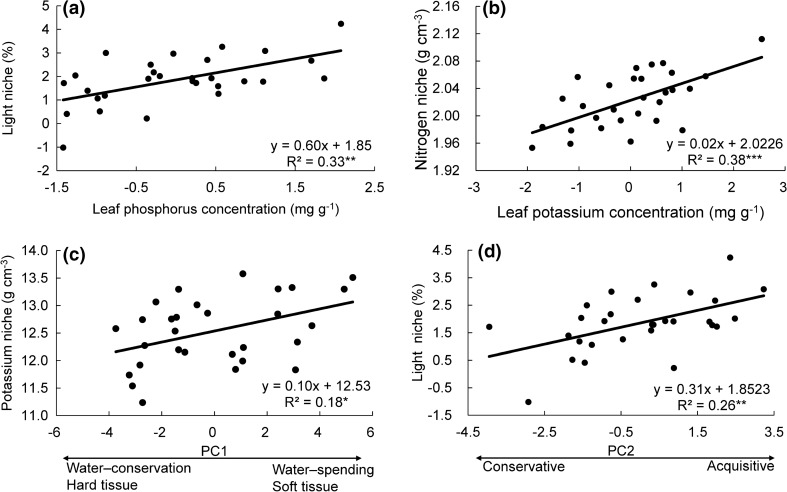
Relationships between **a** light niche vs leaf phosphorus concentration, **b** nitrogen niche vs leaf potassium concentration, **c** phosphorus niche vs trait PC1 (a strategy axis of growth efficiency and water use, see Fig. 1), and **d** light niche vs trait PC2 (a strategy axis of nutrient and carbon acquisition, see Fig. 1). Regression lines, regression equations, R^2^ and significance level (*P < 0.05; **P < 0.01; ***P < 0.001) are shown. Each dot is a species. Resource niches and traits were normalized as described in the “Methods” section

The all-subset regression analysis showed that liana resource niches were determined by different functional traits (Table 3, Fig. 3, and Table S3). For the light niche, the best models (ΔAICc < 2) showed that species associated with higher light conditions had higher LPC and LZnC (Table 3, Fig. 3a). Liana species occupying high water niches (TWI) had high SPI and LPC, but low LNC, vein density (VD) and thin leaves (low LT, Table 3). Liana species that occupied nitrogen richer soils had higher LKC but thin leaves (low LT, Table 3). Species that were found at higher soil phosphorus levels had higher leaf phosphorus and potassium concentrations but lower LNC and LT (Table 3). LPC and LNC were the best predictor of the potassium niche (as indicated by the highest relative importance based on the sum of the Akaike weights). Liana species with high LPC occupied niches with high potassium concentrations, whereas lianas with high LNC occupied niches with low potassium concentrations (Table 3).

**Table 3:** The average regression models predicting the effects of the functional traits on niche dimensions and species relative abundance, based on all possible subset combinations of all 18 traits

	Light niche (%)		Water niche (TWI)		Nitrogen niche (g cm^–3^)		Phosphorus niche (g cm^–3^)		Potassium niche (g cm^–3^)		Relative abundance (%)	
	Avg	Imp	Avg	Imp	Avg	Imp	Avg	Imp	Avg	Imp	Avg	Imp
Intercept	1.85	1.00	1.70	1.00	2.02	1.00	0.37	1.00	12.54	1.00	0.49	1.00
LT			**− 0.02**	1.00	**− 0.02**	1.00	**− 0.03**	1.00				
SLA	− 0.18	0.06							0.16	0.46		
SD	0.22	0.07					0.01	0.16			**0.39**	1.00
VD	0.23	0.07	**− 0.02**	1.00			− 0.02	0.54				
SPI	0.29	0.47	**0.01**	0.68								
LNC			**− 0.03**	1.00	− 0.01	0.32	**− 0.02**	0.84	**− 0.28**	1.00		
LPC	**0.42**	0.88	**0.03**	1.00			**0.02**	0.84	**0.40**	1.00	0.16	0.06
LKC					**0.03**	1.00	**0.02**	1.00				
LMgC											− 0.17	0.24
LZnC	**0.40**	0.84							0.22	0.79	− 0.24	0.48
VesD	0.33	0.70									0.23	0.36
SRL											− 0.23	0.45

Traits with high Variance Inflation Factor values (VIF > 5) were removed prior to the test. The average model was calculated for all best models (ΔAICc < 2), the average coefficients (Avg) were presented, and only significant (*P* < 0.05) results were given in bold. Relative importance (Imp) of the predictor variables was calculated as the sum of the Akaike weights over all best models in which the parameter of interest appeared. For trait abbreviations, see Table 1

Liana resource niches can also be predicted by multivariate trait strategies as captured by species scores on the PCA axes (Fig. 1). Linear regression indicated that PC1 was significantly positively related to the liana species distribution along gradients in soil potassium (*R*^2^ = 0.18, *P* = 0.0227, Fig. 3c and Table S4) and tended to be positively related to liana distribution along the topographic water availability (*R*^2^ = 0.11, *P* = 0.0751, Table S4). PC2 was positively related to the potassium (*R*^2^ = 0.22, *P* = 0.0112, Table S4) and light availability (*R*^2^ = 0.26, *P* = 0.0044, Fig. 3d and Table S4) niches. Surprisingly, neither PC1 nor PC2 were significantly related with the water niche (TWI, Table S4).

**Fig. 4:**
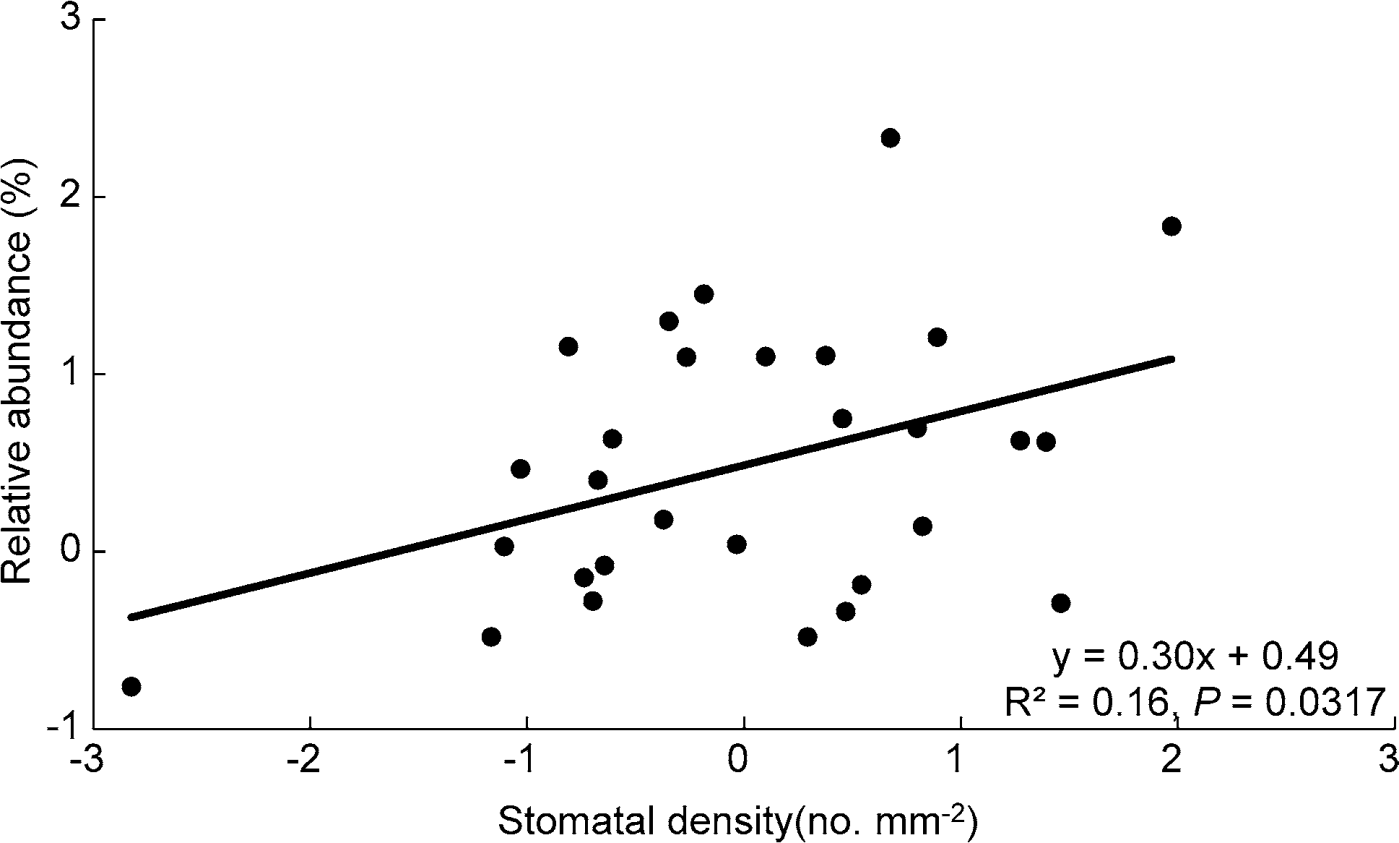
Relationship between relative abundance and stomatal density across 29 liana species in Xishuangbanna tropical seasonal rainforest. Regression lines, regression equations, and R^2^ are shown. Each dot indicates a species. Relative abundance was calculated as the total individuals of each species divided by the total individuals of all liana species across the plot. Variables were normalized as described in the “Methods” section

Liana multivariate trait strategies were not associated with species abundance (Table S4). Surprisingly, stomatal density was the best predictor of liana abundance, with species possessing more stomata attaining a higher abundance (Table 3 and Fig. 4), although this trend was mainly driven by Kadsura heteroclita; no significant relationship between liana abundance and traits was found when the species was excluded.

## Discussion

We evaluated traits from 29 coexisting liana species and asked how liana traits are associated, what plant strategies can be distinguished, and whether different traits shape different species niches. We found that lianas showed a primary spectrum in tissue toughness and water use, and a secondary spectrum in nutrient and carbon acquisition. Liana species with more acquisitive trait values occupied higher light and nutrient resource niches, but different traits were important for different niche dimensions.

### A primary spectrum in toughness and water use, and a secondary spectrum in nutrient and carbon acquisition

We hypothesized that liana species would show a trait spectrum, ranging from trait values that increase resource acquisition and use, to trait values that increase resource conservation. Rather than one spectrum we found two spectra: the first PCA axis represents a tissue toughness and water-spending spectrum, and the second PCA axis a resource acquisition spectrum (Fig. 1). Intriguingly, each spectrum partly reflects the conservative–acquisitive paradigm, but at the same time these two spectra are also independent from each other because they are captured by two orthogonal PCA axes.

#### Spectrum in toughness and water use

The first PCA axis represents a toughness spectrum where traits are aligned along an axis from soft to hard tissues, with thick leaves to the right and high leaf-, wood- and leaf venation density to the left (Fig. 1). This trait association along the first axis underlies the growth-survival trade-off that is frequently observed across tropical liana species (Gilbert et al. 2006) and tree species (Wright et al. 2010). Species either invest in soft tissues that facilitate fast, cost-efficient growth or they invest in hard and persistent tissues that enhance resource conservation and plant survival (Poorter and Bongers 2006; Kitajima and Poorter 2010).

This tissue toughness spectrum also reflects a spectrum in water spending and conservation. At the right, there is a group of traits that are important for hydraulic integration and increased water use, with wide vessels that facilitate high stem hydraulic conductivity (Tyree et al. 1994), thick leaves with a large water content that facilitates water storage (Camilleri and Ribi 1983), large stomata that facilitate stomatal conductance and gas exchange (Lambers et al. 2008), and high leaf K concentration which is used in the guard cells to fine-tune stomatal aperture in response to temporal variation in environmental conditions (Benlloch-González et al. 2008).

At the left hand of the PCA axis, there are two hydraulic traits (leaf venation density and stomatal density) that align with tough tissues. A high stomatal density allows plants to regulate water loss more precisely (Lawson and Blatt 2014), thus improving water conservation. Normally, vein density is thought to be associated with high water transport capacity and high photosynthetic carbon gain (Sack et al. 2005) but the fact that it does not align with the leaf photosynthetic spectrum (PCA Axis 2) means that vein density fulfills a different role. Veins have a dual function; they are not only important for water transport, but also they increase the structure, hardiness, and longevity of the leaf (Kitajima and Poorter 2010).

#### Spectrum in nutrient and carbon acquisition

The second axis represents a nutrient and carbon acquisition spectrum, with species having a low capacity to acquire soil and light resources at the bottom and species with a high capacity to acquire and use resources at the top (Fig. 1); high specific root length facilitates nutrient uptake (Eissenstat 1992; Eissenstat et al. 2000), large leaves and high specific leaf area facilitate light capture (Reich et al. 1998), and high nitrogen and phosphorus concentrations allow for investments in photosynthetic proteins and ATP that increase photosynthetic capacity and carbon gain (Reich et al. 2003).

Although this second axis closely reflects the leaf economics spectrum (Wright et al. 2004; Onoda et al. 2017), it has one important difference. It only reflects resource acquisition and use but not resource conservation, as it is not related to tough and persistent tissues such as high leaf density or wood density (Fig. 1). Therefore, this nutrient and carbon acquisition spectrum is orthogonal to the tissue toughness spectrum.

In our case, the two axes are decoupled because of the traits that underlie specific leaf area (SLA). SLA plays a pivotal role in the leaf economics spectrum, as high SLA facilitates resource capture and use, and low SLA and its underlying traits (high leaf thickness and/or high leaf density) facilitate resource conservation. In our study, SLA is equally determined by leaf thickness and leaf density. Leaf density and its analogue, leaf dry matter content, are in our case associated with PC1 (Fig. 1), and have a stronger impact on leaf toughness, leaf longevity (Kitajima and Poorter 2010; Kitajima et al. 2012) and nutrient conservation (Hodgson et al. 2011) than SLA, which in our case is associated with PC2. This explains why in our case, the leaf economics spectrum, where all traits are assumed to be linked to the same axis (Wright et al. 2004), falls apart into two spectra.

#### Are root–stem and leaf traits coupled?

We hypothesized that liana stem traits can be decoupled from leaf and root traits, because lianas are structural parasites, investing fewer resources in structural support of the stem. We found that liana stem toughness (wood density) aligned with leaf toughness (leaf density and dry matter content; Table 2, Fig. 1), indicating a life history coordination across organs. We also found that liana stem water transport capacity (maximum vessel diameter) aligned with leaf water transpiring capacity (stomatal size; Table 2, Fig. 1), indicating a hydraulic integration across stem and leaf organs. In contrast, Baraloto et al. (2010) found that leaf economics spectrum and stem economics spectrum were orthogonal in Neotropical trees, suggesting that trade-offs operate independently at the leaf and at the stem levels.

The only root trait that we analyzed (specific root length) was not significantly correlated with any of the stem and leaf traits (Table 2), which is in line with the suggestion of Weemstra et al. (2016) that roots are not associated with the plant economics spectrum, as they have to acquire many different nutrient resources, and they can acquire them in many different ways (e.g., through mycorrhizae or root exudates).

### Liana species with acquisitive trait values occupy higher resource niches

We hypothesized that liana species with more acquisitive trait values occupy higher light, water, and nutrient resource niche dimensions. We found that different functional traits shaped different niche dimensions (Table 3) and that multivariate trait strategies also play a role in shaping liana distribution (the first PCA axis had a significant positive effect on liana soil niches, Fig. 3c). This indicates that liana species with more acquisitive trait values (softer tissues, greater water use) can take advantage of these conditions and dominate high soil resource niches.

When resource niches were predicted based on individual traits, then acquisitive trait values indeed often increased the resource niche (i.e., P, K, Zn, stomatal pore index, and thinner leaves increased different resource niches), but not always (e.g., a high leaf nitrogen concentration decreased the soil water, P, and K niche, Table 3). Plant strategies are inherently multivariate and thought to better explain the species niche (Grime 2006). Yet, in our case, individual traits were better predictors of the species niche dimensions than the multivariate strategy axes (i.e., the R^2^ was higher; Table S3 and Table S4). This indicates that different components of the multivariate strategy axes, rather than the main strategies themselves are important for different niche dimensions, although it could be, of course, that additional PCA axes, and less obvious axes of plant trait variation, could explain additional variation.

#### Light niche

We hypothesized that the light niche of lianas would increase with traits that increase carbon gain, for example, through increased light capture ability (large leaf area and high specific leaf area), high leaf N and P concentrations, and fast gas exchange (high stomatal density, length, and pore index). We indeed found that the light niche was predicted by the multivariate nutrient and carbon acquisition axis (PC2; Fig. 3d and Table S4). The light niche mainly increased with leaf phosphorus and zinc concentrations, and to a lesser extent with vessel density and stomatal pore index (which have a large relative importance; Table 3 and Table S3). Leaf zinc concentration has rarely been studied in tropical rainforests. Zinc helps with the production of a plant hormone responsible for stem elongation and leaf expansion (Lines-Kelly 1992), which should especially be important for lianas with their climbing life form. Light-demanding lianas tend to have wide vessels that increase the water transport capacity of the stem (van der Sande et al. 2019), thus allowing for fast gas exchange. Similarly, a high stomatal pore index allows for a high stomatal conductance and gas exchange (Bidwell 1974) to optimally benefit from the high irradiance.

#### Water niche

We hypothesized that the water niche would be best predicted by water transport traits (i.e., vessel diameter, leaf venation, and stomata). We found that species with large stomatal pore index (i.e., high gas exchange), thin leaves that desiccate easily and large leaf P occupied topographically wet habitats. Species with dense leaf venation and large leaf N occupied dry habitats, for which we do not have a clear explanation (Table 3). Other studies have shown that within the same community, the topographic water niche is determined by a suite of traits (Cosme et al. 2017; Oliveira et al. 2019). For example, Amazonian rainforest tree species from higher and relatively drier plateaus had lower SLA, denser wood, narrower vessels, lower hydraulic conductivity, and stronger resistance against drought-induced cavitation than species from lower-lying wet valleys (Cosme et al. 2017; Oliveira et al. 2019). We used a rather coarse measure (topographic water availability) to quantify the water niche. Future studies should really measure soil moisture at different soil depths to increase our understanding of the water niche. Nevertheless, water is probably not a strongly limiting factor in our moist and shaded forest; in Xishuangbanna, fog drip contributes 5% of the annual rainfall, with 86% of the fog drip occurring in the dry season, thus alleviating the effect of seasonal drought (Liu et al. 2004).

#### Nutrient niche

We hypothesized that liana nutrient niches would increase with traits that reflect nutrient requirements and use and especially with leaf P because P is often limiting in old weathered and leached tropical soils (Vitousek et al. 2010). Soil nutrient niches were indeed closely associated with leaf nutrient concentrations; soil nutrient niches increased with leaf P (for soil P and K), and leaf K (for soil P and N), and decreased with leaf N (for soil P and K; Table 3, Fig. 3b). In addition, soil K niches could also be predicted by the multivariate tissue toughness spectrum (Table S4, Fig. 3c), with species bearing softer tissues occupying higher resource niches. Species with tough and persistent tissues can retain nutrients for a longer time in their leaves and branches, and as a result have lower nutrient requirements, and can better persist under low soil resource conditions (Aerts 1996).

### Functional traits were not associated with liana abundance

We hypothesized that in this humid, light-limited forest, conservative trait values that increase shade tolerance (e.g., high wood density and low specific leaf area) would increase the abundance of liana species. Surprisingly, none of these traits had a significant effect on liana abundance (Table 3, Fig. 4, Table S3), despite the fact that we included several traits belonging to the leaf economics spectrum and stem economics spectrum that are thought to be generally important for plant strategies and functioning (Wright et al. 2004; Chave et al. 2009). Previous studies have shown that under low light conditions, tree species with conservative trait values such as low SLA, high wood density and leaf dry matter content attain higher abundance at the sapling stage (Reich et al. 1997; Cornwell and Ackerly 2010) because they can retain their hardly acquired carbon for longer periods of time. Similarly, in Panama, these conservative traits are able to predict the abundance of trees, but not of lianas (van der Sande et al. 2019). Perhaps in this Panamanian study as well as in our study, no relationships between traits and liana abundance were found because relatively large lianas were studied (with a stem diameter > 1 cm) which already have most of their leaves in the forest canopy, and hence, are not light limited. Stronger effects of light on lianas might be expected in the seedling stage, during which more individuals are found in shaded conditions. We found that stomatal density tended to shape liana abundance (Table 3). Denser but smaller stomata may allow for a better control of gas exchange during drought or sun flecks (Düring 2015; Voelker et al. 2016).

### How functional are functional traits?

Plant functional traits and strategies can indeed explain species distribution, but not in a simple and straightforward way as we hoped for. This research shows that (1) global trait economics spectra can also be found in local plant communities, but part of these trait economics spectra can be uncoupled, (2) it is the underlying components (i.e., individual traits), rather than plant strategies (i.e., overall trait syndromes) themselves that determine the species niche, and (3) different traits are important for different niches. Although identifying global plant strategies has significantly advanced the field, this study shows that global, multivariate generalizations are difficult to translate into local conditions, as different components of these strategies may be important under different local conditions.

This study brings us back to the key question about the functionality, validity, and predictability of the ‘functional ecology approach’. Perhaps the field of functional ecology faces such a strong tension between generalization versus contextualization because functionality is, by definition, context dependent. This tension makes the field not only more complicated, but also more interesting and exciting.

Future studies could include more process-based traits (e.g., cavitation vulnerability and leaf specific conductivity) or whole-plant traits (e.g., biomass allocation), and especially root traits (e.g., rooting depth, mycorrhizae, root exudates) to unravel links between traits and soil water and nutrient niches. Similarly, future studies could quantify the species niche by taking spatial autocorrelation into account (cf. Harms et al. 2001).

## Conclusions

We evaluated the functional trait associations and strategies among 29 lianas species, and the correlations between resource niches and functional traits. Lianas showed two orthogonal trait spectra, from tissue toughness and water conservation to tissue softness and rapid water acquisition, and a secondary spectrum in nutrient and carbon acquisition. Liana species with more acquisitive trait values occupied higher light, water and nutrient resource niches, but different traits were important for different niche dimensions. Instead of local plant abundance, traits may better explain species distributions and their presence along gradients of resource availability.

## Supplementary Information

Below is the link to the electronic supplementary material.

Supplementary file1 (DOCX 341 KB)

## Data Availability

Species mean trait data are available from the TRY database, and data on species traits and niches are available from Data Archiving and Networked Services (DANS): https://doi.org/10.17026/dans-xej-j7kf
