# Supplementary material for: Traits, strategies, and niches of liana species in a tropical seasonal rainforest
Source: Oecologia. 2021 May 23;196(2):499–514. doi: 10.1007/s00442-021-04937-4 (PMC8241640; doi:10.1007/s00442-021-04937-4)
Supplement: Supplementary file 1 — Supplementary file1 (DOCX 341 KB) [file 442_2021_4937_MOESM1_ESM.docx]

**Supplementary material**

**Table S1** Overview of the 29 dominant liana species in the Xishuangbanna 20–ha tropical seasonal rainforest dynamics plot: species, code, abundance (total individuals in the 20–ha plot with diameter at breast height ≥ 1 cm), individuals sampled and loadings of first two axes of principal component analysis (PCA) using specific mean values as data points

| Species | Code | Abundance | Samples | PC1 | PC2 |
| --- | --- | --- | --- | --- | --- |
| *Parameria laevigata* | Pa_la | 2386 | 12 | -1.49 | -1.59 |
| *Byttneria aspera* | By_as | 1452 | 11 | -2.22 | 1.81 |
| *Fissistigma polyanthum* | Fi_po | 990 | 10 | -1.13 | -0.77 |
| *Gnetum montanum* | Gn_mo | 849 | 8 | 1.07 | -0.94 |
| *Combretum latifolium* | Co_la | 776 | 3 | -1.63 | 0.31 |
| *Sargentodoxa cuneata* | Sa_cu | 736 | 4 | 3.07 | -0.45 |
| *Uvaria kweichowensis* | Uv_kw | 698 | 5 | -0.66 | 0.35 |
| *Strychnos angustiflora* | St_an | 694 | 10 | -2.65 | -1.88 |
| *Spatholobus uniauritus* | Sp_un | 693 | 6 | 1.08 | 1.96 |
| *Callerya pachyloba* | Ca_pa | 490 | 6 | -3.74 | 1.88 |
| *Benkara sinensis* | Be_si | 464 | 10 | -2.73 | -1.45 |
| *Tetrastigma cauliflorum* | Te_ca | 437 | 13 | 2.95 | -0.07 |
| *Uncaria macrophylla* | Un_ma | 433 | 11 | -1.44 | 1.31 |
| *Uncaria laevigata* | Un_la | 430 | 9 | -2.73 | -0.75 |
| *Tetrastigma planicaule* | Te_pl | 369 | 7 | 4.93 | 0.29 |
| *Tetrastigma jinghongense* | Te_ji | 346 | 11 | 2.40 | -1.39 |
| *Embelia undulata* | Em_un | 277 | 10 | 0.68 | -2.92 |
| *Premna scandens* | Pr_sc | 267 | 9 | -1.36 | 2.48 |
| *Dalbergia stipulacea* | Da_st | 241 | 13 | -3.11 | 0.88 |
| *Tetracera sarmentosa* | Te_sa | 238 | 7 | 0.80 | -1.77 |
| *Tetrastigma obovatum* | Te_ob | 214 | 7 | 5.25 | 2.35 |
| *Tetrastigma xishuangbannaense* | Te_xi | 200 | 4 | 3.15 | 0.37 |
| *Capparis fohaiensis* | Ca_fo | 193 | 8 | 1.10 | 0.87 |
| *Uvaria tonkinensis* | Uv_to | 175 | 3 | -1.36 | 0.66 |
| *Strychnos nitida* | St_ni | 173 | 8 | -3.24 | -1.27 |
| *Zanthoxylum laetum* | Za_la | 165 | 6 | -0.26 | 3.23 |
| *Piper flaviflorum* | Pi_fl | 143 | 11 | 2.42 | 2.00 |
| *Roureopsis emarginata* | Ro_em | 143 | 6 | -2.83 | -1.54 |
| *Kadsura heteroclita* | Ka_he | 108 | 3 | 3.70 | -3.95 |

**Table S2** The trait loadings of first two axes of principal component analysis (PCA).

| Traits | Abbreviation | PC1 | PC2 |
| --- | --- | --- | --- |
| Leaf thickness | LT | 0.30 | -0.16 |
| Leaf area | LA | 0.18 | 0.33 |
| Leaf density | LD | -0.34 | -0.03 |
| Leaf dry-matter content | LDMC | -0.35 | -0.02 |
| Specific leaf area | SLA | 0.01 | 0.30 |
| Vein density | VD | -0.33 | 0.10 |
| Stomatal density | SD | -0.28 | 0.18 |
| Stomatal length | SL | 0.30 | -0.19 |
| Stomatal pore index | SPI | 0.05 | 0.03 |
| Leaf nitrogen concentration | LNC | -0.07 | 0.42 |
| Leaf phosphorus concentration | LPC | 0.15 | 0.43 |
| Leaf potassium concentration | LKC | 0.24 | 0.21 |
| Leaf magnesium concentration | LMC | 0.17 | -0.08 |
| Leaf zinc concentration | LZC | -0.01 | 0.43 |
| Leaf nitrogen to phosphorus ratio | N:P | -0.26 | -0.09 |
| Wood density | WD | -0.32 | -0.11 |
| Maximum vessel diameter | VesD | 0.27 | -0.06 |
| specific root length | SRL | 0.01 | 0.28 |

**Table S3** Regression models predicting the effects of the functional traits on niche dimensions and species relative abundance, based on all possible subset combinations of all 18 traits. Traits with high Variance Inflation Factor values (VIF > 5) were removed prior to the test. For each resource niche, coefficients of all best models (ΔAICc < 2) were presented, and only significant (*P* < 0.05) results were given in bold. Per model, degree of freedom (df), the log likelihood (logLik), corrected Akaike information criterion (AICc), the AICc weight, adjusted R^2^ and *P*-value were given. Models were ordered from the best to poorest, based on the AICc’s. For trait abbreviations, see Table S2.

Light niche

| Model | (Intercept) | LPC | LZnC | VesD | SD | SLA | SPI | VD | df | logLik | AICc | delta | weight | R^2^ | *P* |
| --- | --- | --- | --- | --- | --- | --- | --- | --- | --- | --- | --- | --- | --- | --- | --- |
| 617 | 1.85 | 0.32 | **0.43** | 0.29 |  |  | 0.27 |  | 6 | -30.84 | 77.49 | 0.00 | 0.17 | 0.45 | <0.01 |
| 105 | 1.85 | **0.38** | 0.36 | 0.31 |  |  |  |  | 5 | -32.62 | 77.85 | 0.36 | 0.14 | 0.40 | <0.01 |
| 553 | 1.85 | **0.41** | 0.35 |  |  |  | 0.29 |  | 5 | -32.78 | 78.17 | 0.67 | 0.12 | 0.40 | <0.01 |
| 609 | 1.85 |  | **0.59** | **0.37** |  |  | **0.32** |  | 5 | -32.84 | 78.30 | 0.80 | 0.12 | 0.39 | <0.01 |
| 9 | 1.85 | **0.60** |  |  |  |  |  |  | 3 | -35.87 | 78.70 | 1.20 | 0.09 | 0.31 | <0.01 |
| 41 | 1.85 | **0.48** | 0.27 |  |  |  |  |  | 4 | -34.63 | 78.93 | 1.43 | 0.08 | 0.34 | <0.01 |
| 2153 | 1.85 | **0.42** | 0.34 | **0.44** |  |  |  | 0.23 | 6 | -31.72 | 79.27 | 1.77 | 0.07 | 0.42 | <0.01 |
| 233 | 1.85 | **0.38** | 0.33 | **0.43** | 0.22 |  |  |  | 6 | -31.79 | 79.40 | 1.90 | 0.07 | 0.41 | <0.01 |
| 73 | 1.85 | **0.55** |  | 0.22 |  |  |  |  | 4 | -34.89 | 79.44 | 1.94 | 0.07 | 0.33 | <0.01 |
| 873 | 1.85 | 0.32 | **0.50** | 0.30 |  | -0.18 | 0.27 |  | 7 | -30.07 | 79.47 | 1.97 | 0.06 | 0.46 | <0.01 |

Water niche

| Model | (Intercept) | LNC | LPC | LT | SPI | VD | df | logLik | AICc | delta | weight | R^2^ | *P* |
| --- | --- | --- | --- | --- | --- | --- | --- | --- | --- | --- | --- | --- | --- |
| 2589 | 1.70 | **-0.03** | **0.03** | **-0.03** | **0.01** | **-0.02** | 7.00 | 70.92 | -122.50 | 0.00 | 0.68 | 0.49 | <0.01 |
| 2088 | 1.70 | **-0.03** | **0.03** | **-0.03** |  | **-0.01** | 6.00 | 68.39 | -120.95 | 1.55 | 0.32 | 0.41 | <0.01 |

Soil N niche

| Model | (Intercept) | LKC | LNC | LT | df | logLik | AICc | delta | weight | R^2^ | *P* |
| --- | --- | --- | --- | --- | --- | --- | --- | --- | --- | --- | --- |
| 18 | 2.02 | **0.03** |  | **-0.02** | 4 | 62.73 | -115.79 | 0.00 | 0.68 | 0.47 | <0.01 |
| 22 | 2.02 | **0.03** | -0.01 | **-0.02** | 5 | 63.44 | -114.27 | 1.52 | 0.32 | 0.48 | <0.01 |

Soil P niche

| Model | (Intercept) | LKC | LNC | LPC | LT | SD | VD | df | logLik | AICc | delta | weight | R^2^ | *P* |
| --- | --- | --- | --- | --- | --- | --- | --- | --- | --- | --- | --- | --- | --- | --- |
| 2078 | 0.37 | **0.02** | **-0.02** | **0.02** | **-0.03** |  | -0.02 | 7 | 66.17 | -113.00 | 0.00 | 0.39 | 0.51 | <0.01 |
| 30 | 0.37 | **0.02** | **-0.02** | **0.02** | **-0.02** |  |  | 6 | 64.15 | -112.47 | 0.53 | 0.30 | 0.46 | <0.01 |
| 18 | 0.37 | **0.03** |  |  | **-0.01** |  |  | 4 | 60.45 | -111.23 | 1.77 | 0.16 | 0.36 | <0.01 |
| 2206 | 0.37 | **0.02** | **-0.02** | **0.02** | **-0.03** | 0.01 | **-0.02** | 8 | 67.19 | -111.18 | 1.83 | 0.16 | 0.53 | <0.01 |

Soil K niche

| Model | (Intercept) | LNC | LPC | LZnC | SLA | df | logLik | AICc | delta | weight | R^2^ | *P* |
| --- | --- | --- | --- | --- | --- | --- | --- | --- | --- | --- | --- | --- |
| 45 | 12.54 | **-0.25** | **0.37** | **0.23** |  | 5 | -18.24 | 49.09 | 0.00 | 0.54 | 0.39 | <0.01 |
| 301 | 12.54 | **-0.32** | **0.40** | 0.19 | 0.13 | 6 | -17.41 | 50.63 | 1.55 | 0.25 | 0.40 | <0.01 |
| 269 | 12.54 | **-0.30** | **0.47** |  | 0.19 | 5 | -19.19 | 51.00 | 1.91 | 0.21 | 0.35 | <0.01 |

Relative abundance

| Model | (Intercept) | LMgC | LPC | LZnC | VesD | SD | SRL | df | logLik | AICc | delta | weight | AdjR2 | *P* |
| --- | --- | --- | --- | --- | --- | --- | --- | --- | --- | --- | --- | --- | --- | --- |
| 161 | 0.49 |  |  | -0.25 |  | **0.36** |  | 4.00 | -28.35 | 66.37 | 0.00 | 0.14 | 0.20 | 0.02 |
| 1217 | 0.50 |  |  |  | 0.25 | **0.51** | -0.27 | 5.00 | -27.20 | 67.00 | 0.63 | 0.10 | 0.23 | 0.02 |
| 1153 | 0.50 |  |  |  |  | **0.36** | -0.24 | 4.00 | -28.69 | 67.04 | 0.67 | 0.10 | 0.18 | 0.03 |
| 225 | 0.49 |  |  | -0.24 | 0.21 | **0.47** |  | 5.00 | -27.32 | 67.26 | 0.88 | 0.09 | 0.23 | 0.02 |
| 129 | 0.49 |  |  |  |  | **0.30** |  | 3.00 | -30.19 | 67.34 | 0.97 | 0.08 | 0.13 | 0.03 |
| 1185 | 0.49 |  |  | -0.20 |  | **0.39** | -0.18 | 5.00 | -27.51 | 67.63 | 1.26 | 0.07 | 0.22 | 0.03 |
| 163 | 0.49 | -0.15 |  | -0.22 |  | **0.32** |  | 5.00 | -27.65 | 67.92 | 1.54 | 0.06 | 0.21 | 0.03 |
| 131 | 0.49 | -0.19 |  |  |  | 0.26 |  | 4.00 | -29.13 | 67.93 | 1.56 | 0.06 | 0.16 | 0.04 |
| 169 | 0.49 |  | 0.16 | **-0.32** |  | **0.38** |  | 5.00 | -27.67 | 67.94 | 1.57 | 0.06 | 0.21 | 0.03 |
| 1249 | 0.49 |  |  | -0.18 | 0.24 | **0.53** | -0.21 | 6.00 | -26.09 | 67.99 | 1.62 | 0.06 | 0.26 | 0.02 |
| 193 | 0.49 |  |  |  | 0.21 | **0.42** |  | 4.00 | -29.20 | 68.07 | 1.70 | 0.06 | 0.15 | 0.04 |
| 1219 | 0.50 | -0.17 |  |  | 0.25 | **0.46** | -0.25 | 6.00 | -26.14 | 68.09 | 1.72 | 0.06 | 0.26 | 0.02 |
| 1155 | 0.49 | -0.17 |  |  |  | **0.32** | -0.22 | 5.00 | -27.77 | 68.16 | 1.78 | 0.06 | 0.20 | 0.03 |

**Table S4** Regression of niches of light, water, nitrogen, phosphorus and potassium and liana relative abundance against PCA components (PCA1 and PCA2). Only significant results were given in bold.

| Variables | PC1 |  | PC2 |
| --- | --- | --- | --- |
|  | R^2^ |  | R^2^ |
| Light niche (%) | 0.09 |  | **0.26** |
|  |  |  |  |
| Water niche (TWI) | 0.11 |  | 0.03 |
|  |  |  |  |
| Nitrogen niche (g cm^–3^) | 0.06 |  | 0.13 |
|  |  |  |  |
| Phosphorus niche (g cm^–3^) | 0.06 |  | 0.13 |
|  |  |  |  |
| Potassium niche (g cm^–3^) | **0.18** |  | **0.22** |
|  |  |  |  |
| Relative abundance (%) | 0.06 |  | < 0.01 |
|  |  |  |  |

**Fig. S1** Relative frequency of 6 environmental variables in the Xishuangbanna season tropical rainforest: a) elevation, b) slope, c) topographic wet index, d) soil N concentration, e) soil P concentration, f) soil K concentration. The histograms were based on the values of 500 20 m x 20 m quadrats within the 20–ha forest plot. Nutrient concentrations were expressed per unit soil volume

**
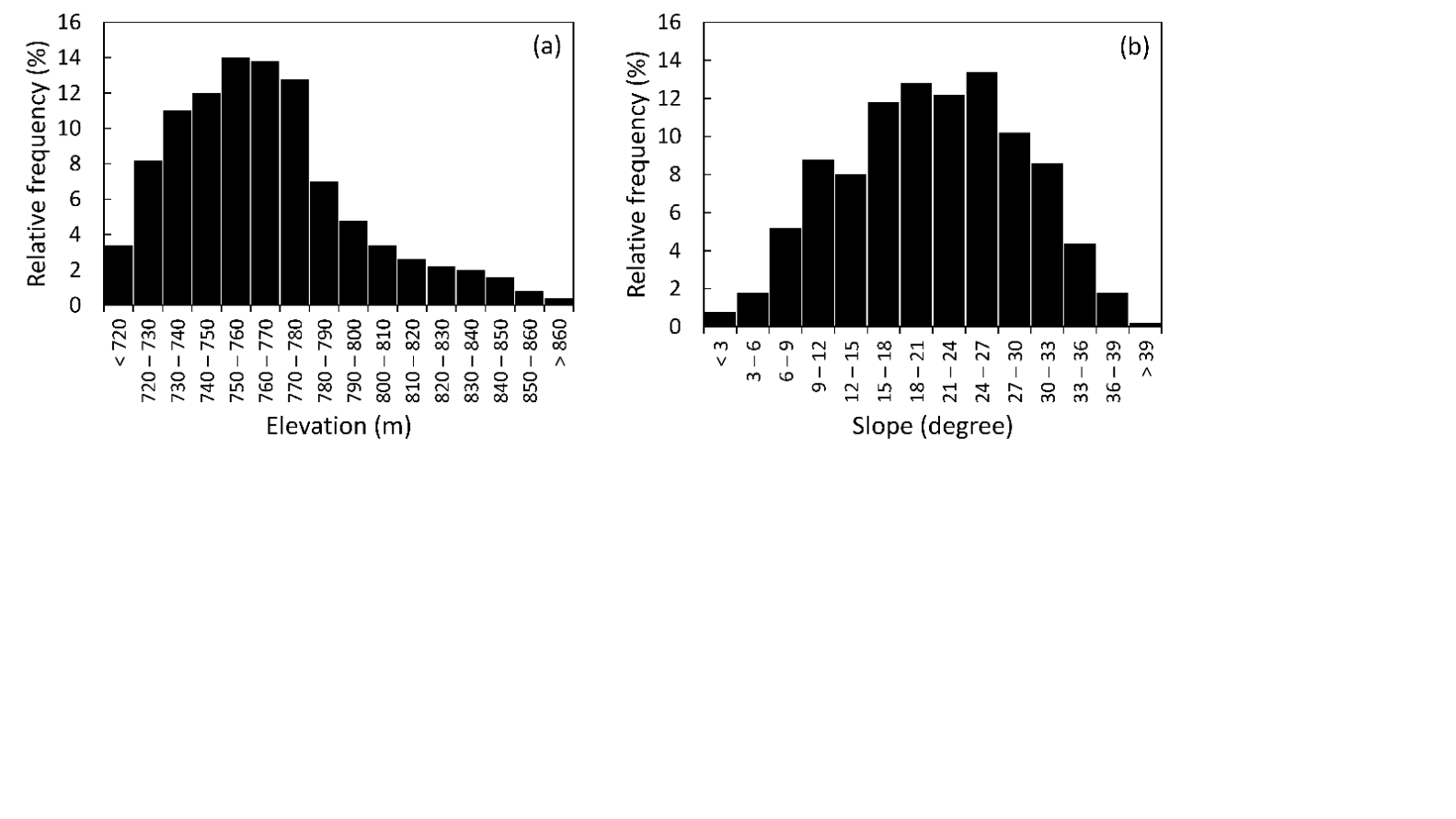
**


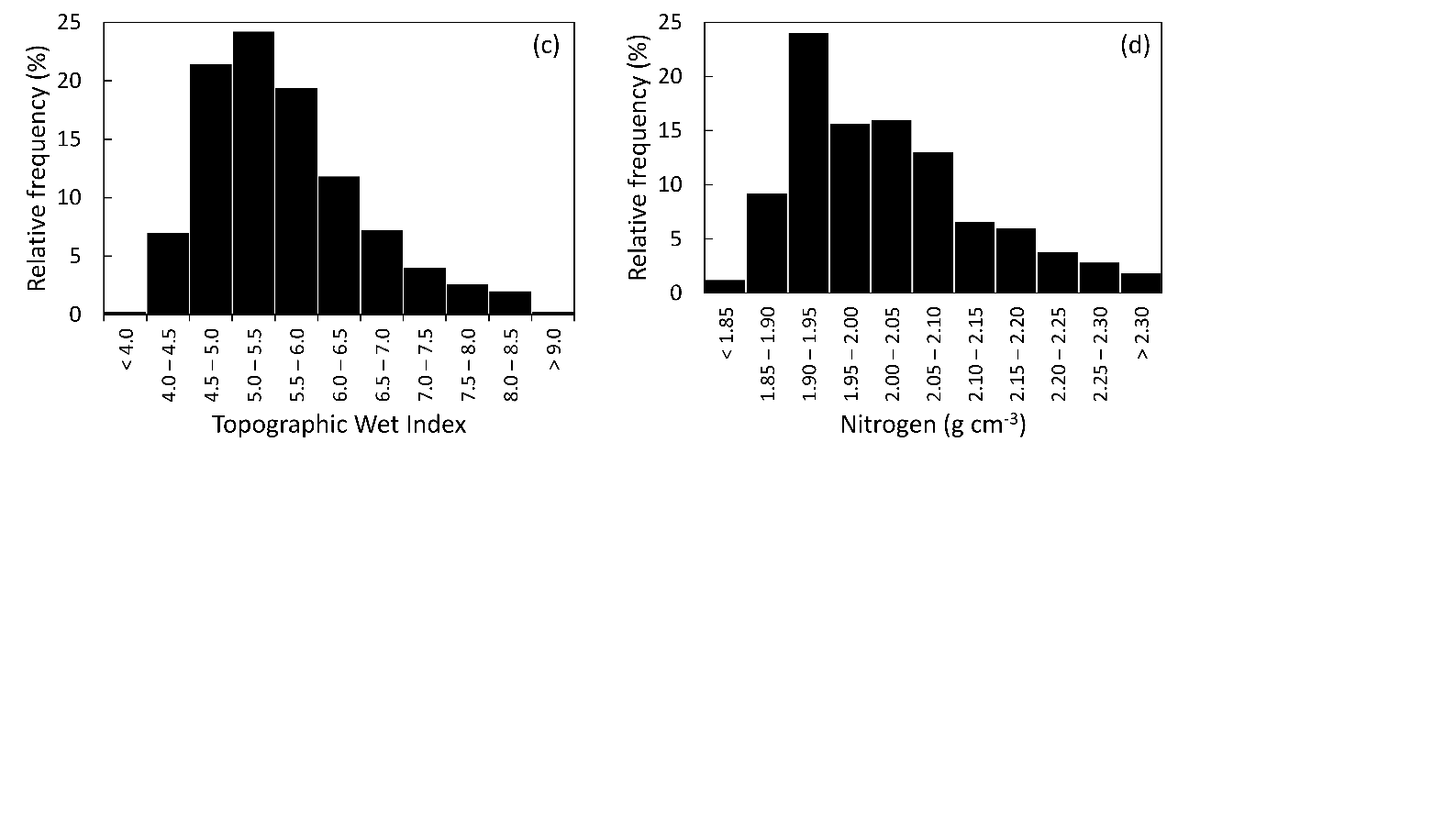


**
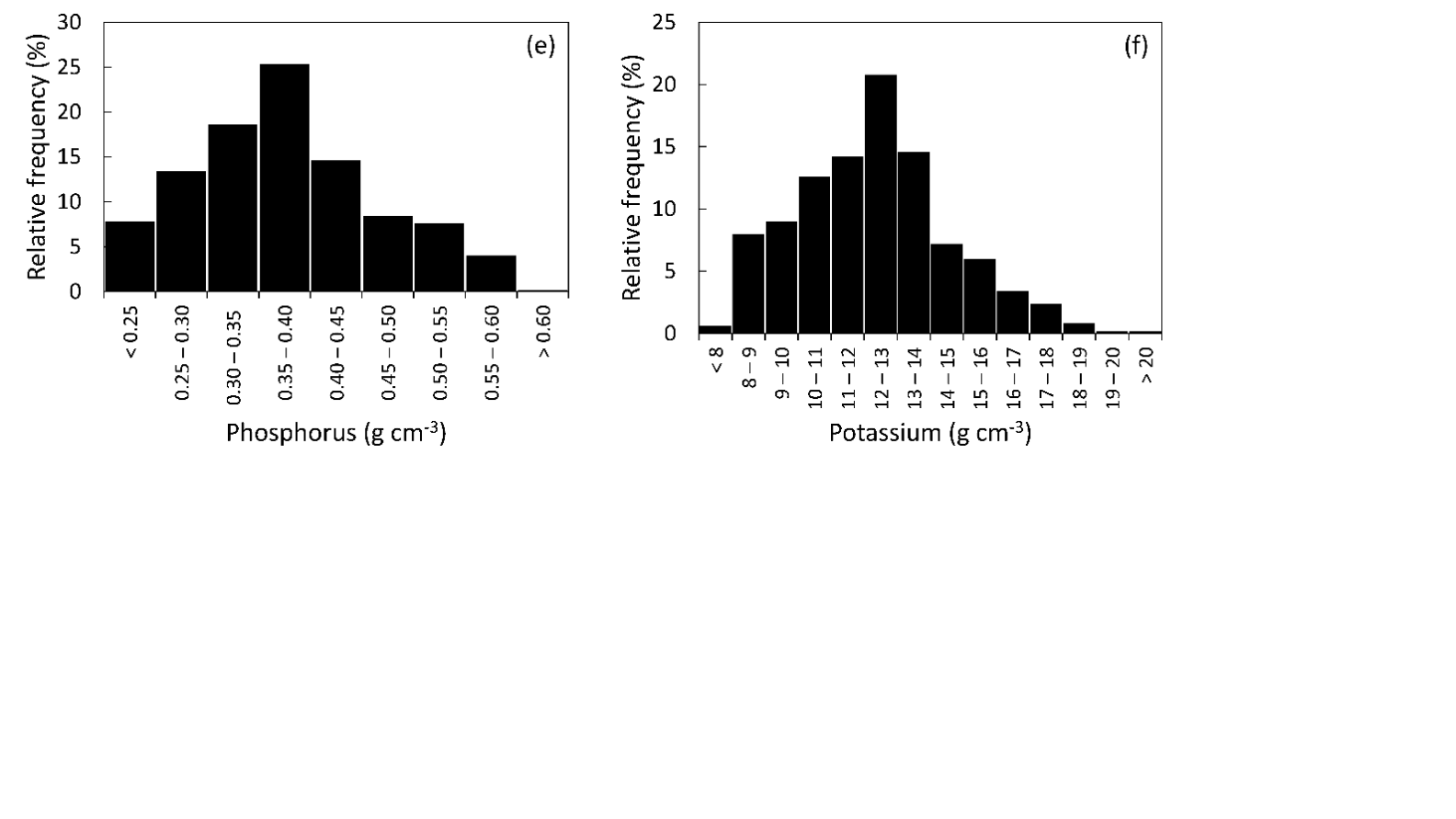
**
